# John H. Griscom

**Published:** 1881-07

**Authors:** 


JOHN H. GRISCOM.—A native of New York and a son, we believe, of the prominent and highly respectable Dr. Griscom of the same city, has been *Tannerising* in Chicago, with results, we presume, so far (June 15th) satisfactory to himself at least. Fasting for forty days seems to have developed into a sort of monomania since Dr. Tanner's useless exposure of his life in that direction a few months ago, and while Mr. Griscom may be possessed of equal physical endurance, and may pass as successfully as he did through the long and trying ordeal of fasting ; it is by no means evident to us, that his prolonged fast will be productive of more fruitful results to science and humanity, than Dr. Tanner's yielded at the expiration of *his* forty days. However, *nous verrons.*

